# Novel metrics for quantifying bacterial genome composition skews

**DOI:** 10.1186/s12864-018-4913-5

**Published:** 2018-07-11

**Authors:** Lena M. Joesch-Cohen, Max Robinson, Neda Jabbari, Christopher G. Lausted, Gustavo Glusman

**Affiliations:** 1 Institute for Systems Biology, 401 Terry Ave N, Seattle, WA 98109 USA; 2 Brown University, Providence, RI 02912 USA

**Keywords:** Nucleotide skew, Leading strand, Lagging strand, Obligate intracellular, Compositional bias, Genome metrics, Lyme disease

## Abstract

**Background:**

Bacterial genomes have characteristic compositional skews, which are differences in nucleotide frequency between the leading and lagging DNA strands across a segment of a genome. It is thought that these strand asymmetries arise as a result of mutational biases and selective constraints, particularly for energy efficiency. Analysis of compositional skews in a diverse set of bacteria provides a comparative context in which mutational and selective environmental constraints can be studied. These analyses typically require finished and well-annotated genomic sequences.

**Results:**

We present three novel metrics for examining genome composition skews; all three metrics can be computed for unfinished or partially-annotated genomes. The first two metrics, (dot-skew and cross-skew) depend on sequence and gene annotation of a single genome, while the third metric (residual skew) highlights unusual genomes by subtracting a GC content-based model of a library of genome sequences. We applied these metrics to 7738 available bacterial genomes, including partial drafts, and identified outlier species. A phylogenetically diverse set of these outliers (i.e., Borrelia, Ehrlichia, Kinetoplastibacterium, and Phytoplasma) display similar skew patterns but share lifestyle characteristics, such as intracellularity and biosynthetic dependence on their hosts.

**Conclusions:**

Our novel metrics appear to reflect the effects of biosynthetic constraints and adaptations to life within one or more hosts on genome composition. We provide results for each analyzed genome, software and interactive visualizations at http://db.systemsbiology.net/gestalt/skew_metrics.

**Electronic supplementary material:**

The online version of this article (10.1186/s12864-018-4913-5) contains supplementary material, which is available to authorized users.

## Background

Bacterial genomes display significant compositional biases, both in terms of G + C content and in compositional skews, i.e., strand asymmetries in ‘T’ vs. ‘A’ and ‘G’ vs. ‘C’ usage [1]. These biases are proposed to arise from the complex interplay of differential mutation rates and multiple selective constraints [2, 3], particularly for energy efficiency [4], involving the replication, repair, and transcription enzymes. Bacterial chromosomes are replicated in both directions, from the origin of replication site to the terminator site; the “leading” strand is replicated continuously while the “lagging” strand is replicated in segments by different enzymes. Some genes are transcribed in the same direction as they are replicated (“leading strand genes”) while others are transcribed in the reverse direction (“lagging strand genes”). Each enzyme mediates both mutational and selective constraints, resulting in different compositional biases in different replicative, transcriptional and translational contexts [4]. Analyses of skews in each context have the potential to expose multiple compositional constraints and their interactions, and ultimately inform about the DNA repair capacity, metabolism, and lifestyle of the species [5, 6].

Compositional bias and strand asymmetry have been measured and analyzed in a variety of ways and contexts [5, 7]. These methods include the original definitions (GC skew, (G-C)/(G + C); AT skew, (A-T)/(A + T)) [8], slight variants (e.g. G/(G + C)) [1], variants based on the three independent axes of Z Curves (x = R-Y, y = M-K, and z = S-W) [9, 10], ANOVA [11], correspondence analysis of codon bias metrics [12, 13], and competing mutational and selective parameters in an explicit evolutionary model [4], and have involved comparison of leading versus lagging contexts, transcribed versus intergenic regions, and restriction to each codon position.

Early examples of extreme compositional biases and asymmetries were found among species in the family Borreliaceae, tick-borne spirochetes including species causing Lyme disease (genus *Borreliella*, formerly *Borrelia*) as well as relapsing fever (genus *Borrelia*) [14]. Since its discovery in 1982 [15], the *Borreliella burgdorferi* spirochete has been of particular interest in the United States as the primary causative agent of Lyme disease. The sequencing of *B. burgdorferi* B31 in 1997 allowed an in depth exploration of the many intriguing features of the genome of this bacterium, from its small size and unusual structure (one large linear chromosome, several linear and circular plasmids) to its very low G + C content [16].

Significant skews in the third position of codons have been reported on both the leading (increased G and T) and lagging (increased A and C) strands in *B. burgdorferi* [12, 17]. Among the first 43 genomes investigated [1], *B. burgdorferi* had the most extreme difference between leading and lagging strand nucleotide compositions. Both mutation and selection biases, variously induced by replication, transcription and translation constraints, have been suggested to play a role in *B. burgdorferi* [18, 19] and more generally, across all prokaryotes. The loss of some DNA repair genes may also contribute to the low G + C content and heightened skew seen in *B. burgdorferi* [6, 20].

Thanks to the much expanded availability of complete genome sequences of bacterial species, it is now possible to perform large-scale comparative genomics studies [2, 4, 21, 22]. A much larger number of bacterial genomes are in draft form, assembled to different levels of contiguity (contigs, scaffolds) and tentatively annotated using automated pipelines. Most of the existing methods for analyzing compositional biases and skews rely on fully or mostly contiguous genomic sequence and on the availability of precise and detailed annotation of genes and direction of replication; such methods are much less applicable to the study of incomplete draft genomes. Furthermore, existing methods largely assess the skews in individual genomes, without taking advantage of the vast knowledge available on the genomes of other bacterial species.

To address these difficulties, we present here three novel metrics for quantitative analysis of genome skews. Our metrics address dependence of skew metrics on G + C content and focus on the differences between nucleotide usage on the leading versus lagging strands, which underlie interpretation of nucleotide skews in terms of both selective and mutational processes. Our skews are also robust to assembly status and can be computed on incomplete genomes with draft annotation, greatly increasing the range of species that can be analyzed. Using these metrics, we analyzed a large collection of bacterial genomes—both complete and draft. We identified several groups of species and genera as outliers on one or more metrics. These outlier species include many pathogens and tend to have unusual lifestyles, like *B. burgdorferi*.

In addition, we have generated and made publicly available an interactive online resource for exploring the skew metrics for thousands of bacterial genomes, and a tool for generating visualizations of skew plots for any bacterial genome of interest with available annotation.

## Methods

### Genomes studied

We obtained from the National Center for Biotechnology Information (NCBI) the genome sequence (in FASTA format) and current annotation (in General Feature Format, GFF) for 7948 bacterial species. We downloaded the “assembly_summary.txt” file from NCBI’s genome FTP site. This file provided various details on 86,822 genome assemblies including the organism name, RefSeq category (whether the genome considered “reference” for the species, “representative”, or otherwise) and assembly level (whether the genome is considered “completed”, or whether it is “incomplete” - assembled to chromosome, scaffold or contig level). Studying this file, we selected and downloaded:

1. 1581 “completed” genomes, (125 “reference”, 1456 “representative”),
2. 3303 “incomplete” genomes, (2 “reference”, 3301 “representative”), and
3. 3064 additional genomes, not repeating species names from the previous two sets, and prioritizing more advanced levels of completion where multiple assemblies are available for a given species.

For each genome, we included in the analysis all chromosomes, plasmids and sequence contigs at least 100 kb long. We removed from further analysis 210 genome assemblies for which the longest available sequence was shorter than 100 kb. The final set of genomes analyzed included 7738 assemblies.

### Identification of origins of replication and terminator sites

For each sequence (chromosome, plasmid, scaffold and contig) in each genome assembly, we computed likely origins of replication and replication terminator sites using the GC disparity method [23, 24], namely by identifying the minimum and maximum difference between the cumulative count of G and C along the genome. This method works well as long as replichores are long [7] and is independent of gene annotation and arbitrary window sizes; it can also efficiently determine the likely direction of replication for sequence fragments (scaffolds and contigs), whether or not they include an origin of replication or a terminator site (see below for evaluation by simulation). When the resulting origin or terminator site lay within 1% of either end of the sequence, we corrected the location to coincide with the nearest sequence end. Where available, we used existing annotations of origin of replication. We obtained the most recent version of the DoriC database of origins of replication [25]. We compared the locations of origins of replication as predicted using the GC disparity method to those annotated in DoriC and evaluated the discrepancy between the two as a fraction of chromosome size, i.e., 0 for no discrepancy and 0.5 for diametrically opposite annotations. DoriC includes 2733 annotations of origins of replication. For 532 species, there are multiple origins annotated on the same chromosome, in which case we retained the one with lowest discrepancy score, i.e., nearest the location predicted by GC disparity. We observed 85 species (3.87%) with discrepancy larger than 0.25 (red points, Additional file 1: Figure S1) and an additional 88 species (4%) with discrepancy larger than 0.1 (green points, Additional file 1: Figure S1). Since DoriC does not include annotations of terminator sites, when using DoriC annotations for origins of replication we assumed the terminator site to be located 0.5 chromosome lengths away from the origin.

### Segmentation and analysis

We used available gene annotation (in the GFF files) to segment each sequence 100 kb or longer into a series of contiguous and disjoint segments (of variable lengths) which can be genes (including CDS, tRNA, and rRNA) or intergenic segments. We stratified intergenic segments by considering the relative orientations of the flanking genes: between two genes in the same orientation, or between two genes in opposite orientations (“head to head” or “tail to tail”). Infrequently, consecutive gene segments may be annotated as overlapping. We excluded such overlapping segments from computation of skews since they have overlapping and likely contradictory constraints.

We computed for each segment (genic or intergenic) its length, G + C content, GC skew, and TA skew. We further determined for each oriented segment (namely genes and intergenic segments between genes transcribed in the same orientation) whether their orientation is the same or opposite to the direction of replication, i.e., whether they are on the leading or lagging strand, relative to origin and terminator sites predicted as described above. As described below, skew computations are done relative to the sense strand of each transcript, but stratified by whether the transcript is on the leading strand during replication (by Pol III) or the lagging strand (replicated by Pol I).

### Computation of characteristic skews

Given a set of comparable segments in a genome assembly (e.g., all genes on the leading strand), we computed skews (GC skew = (G-C)/(G + C) and TA skew = (T-A)/(T + A)) for the set as the average of the corresponding individual segment skews, weighted by segment length. We thus computed four characteristic skews for each species: lead_GC_ and lead_TA_ for leading strand genes, and lag_GC_ and lag_TA_ for lagging strand genes. We also evaluated weighted medians instead of weighted averages, which yielded very similar results (not shown).

### Computation of the cross-skew and dot-skew

The four characteristic skews for a species can be interpreted as two characteristic skew vectors: one for the leading strand genes (lead_GC_, lead_TA_) and the other for the lagging strand genes (lag_GC_, lag_TA_). We computed the cross-skew as:

1 $$ \mathrm{cross}\hbox{-} \mathrm{skew}\left(\mathrm{lead},\mathrm{lag}\right)=\left|\mathrm{lead}\right|\bullet \left|\mathrm{lag}\right|\bullet \sin \left(\theta \right) $$

where |lead| = sqrt(lead_GC_^2^ + lead_TA_^2^), |lag| = sqrt(lag_GC_^2^ + lag_TA_^2^), and *θ* is the angle between the two vectors. Similarly, we computed the dot-skew as:

2 $$ \mathrm{dot}\hbox{-} \mathrm{skew}\left(\mathrm{lead},\mathrm{lag}\right)=\left|\mathrm{lead}\right|\bullet \left|\mathrm{lag}\right|\bullet \cos \left(\theta \right) $$

### Computation of the residual skew

We modeled each of the four characteristic skews (lead_GC_, lead_TA_, lag_GC_ and lag_TA_) as a function of the G + C content for 7738 bacterial genome assemblies. For each characteristic skew we separated the genome assemblies with G + C content below or above 50% G + C (3635 and 4103 genomes, respectively), and fitted a robust regression line to each subset using the R function MASS::lqs() (least trimmed sum of squares, [26]). We then computed a single skew deviation magnitude metric (the residual skew) for each genome as the root mean square deviation (RMSD) from the regression line across the four characteristic skews.

### Identification of outliers

For dot-skew and cross-skew, we identified outliers at both ends of the distribution using the MAD-Median Rule [27] at a significance threshold of 1%. Residual skew has a non-negative distribution, and only atypically high values are of interest; we observed that distribution of residual skew is well approximated by a χ^2^-distribution with 5.8 degrees of freedom, divided by 100 (5.8/100 is the mean observed residual skew value). We therefore used the 99th percentile of this model distribution (0.1647) as a 1% significance threshold.

### Simulation of draft genomes

Starting from a completed genome sequence, we simulated progressively less finished draft genomes by randomly choosing from 1 to 100 cut sites, subdividing the sequence and annotation into contigs based on the cut sites, and computing all metrics as above. We ignored annotations straddling cut sites and, as above, resulting contigs shorter than 100 kb.

## Results

### Overview of the strategy

**Fig. 1:**
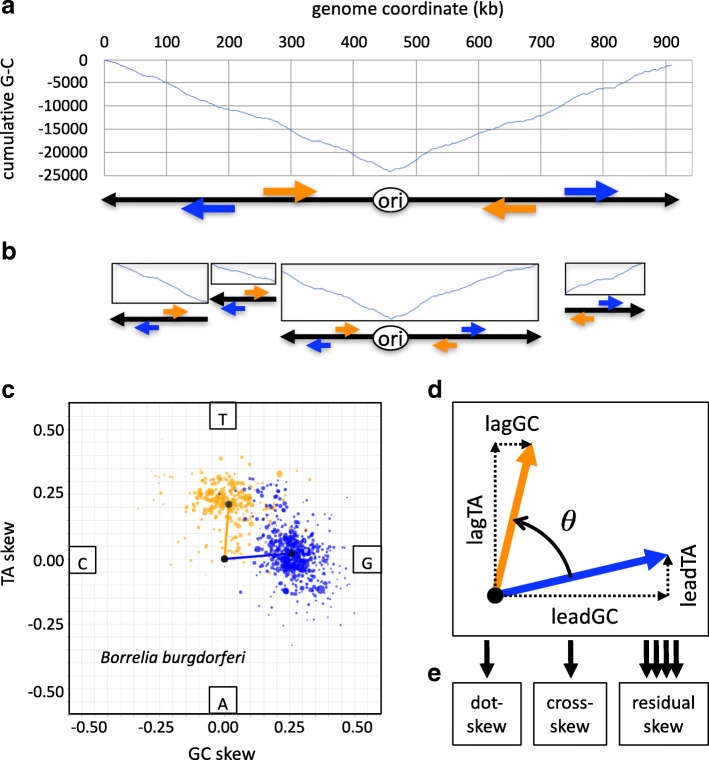
Overview of the method, using *B. burgdorferi* as example. **a**: In the absence of annotated origins of replication, the minimal value of the cumulative G-C graph is used to determine the likely origin of replication (ori) and hence the predicted directions of replication (black arrows) leading to terminator sites (graph maxima). Genes transcribed in those directions (blue arrows) are considered to be on the leading strand, while genes transcribed in the opposite directions (orange arrows) are on the lagging strand. **b**: Treatment of draft genome assemblies. Each contig is analyzed separately to determine likely directions of transcription, from minimal to maximal values of cumulative G-C. Putative origins of replication, terminator sites and gene orientations are determined as above. **c**: For each gene on the leading strand (blue) or lagging strand (orange), TA and GC skews are computed relative to the leading strand. Circle area is proportional to gene length. The vectors point from the origin (zero skews) to the weighted average of skews for genes on the leading strand and genes on the lagging strand. **d**: definition of the characteristic skews (leadGC, leadTA, lagGC and lagTA), and the angle *θ* between the two vectors. **e**: The three metrics computed based on the characteristic skews and the angle *θ*. The multiple arrows leading to the third metric (residual skew) denote that this metric integrates information from many genomes

We have developed a method for analyzing bacterial genome sequences in four main steps. First, we identify the origin of replication (ori) and terminator sites (ter), either using the GC discrepancy method or, where available, existing annotation; these determine the direction of replication (leading or lagging strand) for each segment of the genome (Fig. 1a). For draft genomes, each contig or scaffold is analyzed separately to determine the presence of ori/ter sites and to estimate direction of replication (Fig. 1b). Second, we segment the genome based on gene annotation, classify genes according to their direction of transcription (on the leading strand or on the lagging strand) and compute GC and TA skews for each segment (Fig. 1c). Third, we aggregate the skews of all genes by strand (leading or lagging), compute four characteristic skews and interpret these as two vectors (Fig. 1d). Finally, we compute three skew metrics, either based on the parameters of one genome (dot-skew and cross-skew) or by integrating information from many genomes (residual skew) (Fig. 1e).

### Robustness to fragmentary status of draft sequences

A significant advantage of our method for determining origins of replication and terminator sites based on GC disparity is that it can be applied to finished and draft sequences alike. Since the GC disparity changes nearly monotonously along chromosomes, particularly for highly skewed species (Fig. 1a), it is possible to hypothesize a direction of replication for any fragmentary sequence (Fig. 1b).

We evaluated robustness by simulation: starting from the finished sequences of the large (4.6 Mb) *E. coli* genome and the small (910 kb) *B. burgdorferi* main chromosome, we simulated progressively less finished draft versions of these genomes. The resulting cross-skew and dot-skew metrics were well approximated from simulated draft versions; skew metric variation increased with decreasing simulated genome draft length (Additional file 1: Figure S2). Other skew parameters were also robust to significant fragmentation of the genome (Additional file 1: Figure S3).

### The characteristic skews of *B. burgdorferi* genes

In *B. burgdorferi*, the majority of genes are transcribed in the same direction as they are replicated (‘leading strand genes’, blue in Fig. 1) while some are transcribed in the direction opposite to replication (‘lagging strand genes’, orange in Fig. 1). Leading strand genes tend to display stronger GC skew (Fig. 1c), while lagging strand genes have strong TA skews. In intergenic segments, the two skews tend to be positively correlated (not shown).

**Fig. 2:**
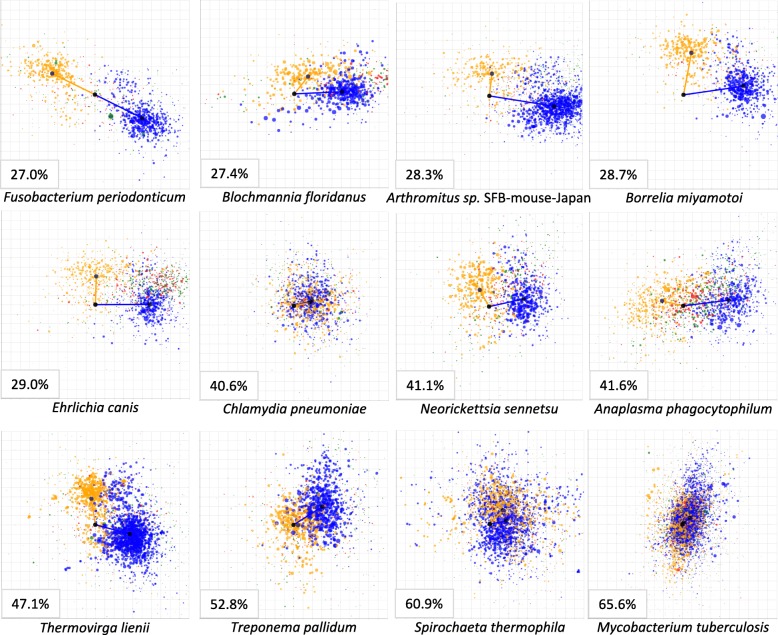
Examples of TA vs. GC skew plots for several bacterial species. Leading strand genes in blue, lagging strand genes in orange, intergenic segments flanked by genes in equal orientation in green, and intergenic segments flanked by genes in opposite orientations in red; circle area is proportional to segment length. Each plot displays skews in the range [− 0.5, 0.5]. Lower-left inset for each plot: average genomic G + C content for that species

Using the strategy delineated above, we computed the four characteristic skews for *B. burgdorferi*: lead_GC_ = 0.258, lead_TA_ = 0.022, lag_GC_ = 0.015 and lag_TA_ = 0.211. These four characteristic skews can also be represented in polar coordinates as two vectors (Fig. 1d). The vector corresponding to leading strand genes has magnitude 0.259 at angle 4.75°, while the vector corresponding to lagging strand genes has magnitude 0.211 at angle 86.08°. In comparison with many other bacterial species (see a few examples in Fig. 2), we observed that such pattern of strong, nearly orthogonal vectors is unusual. For example, we observed small-magnitude vectors in *Spirochaeta thermophila* and *Mycobacterium tuberculosis*, and nearly diametrically opposed vectors in *Fusobacterium periodonticum* and *Anaplasma phagocytophilum*. Some bacterial species (including *Blochmannia floridanus* and *Ehrlichia canis*) had a similar pattern to that observed in *B. burgdorferi*. We discuss these in more detail in subsequent sections.

To quantify the unusual pattern observed in *B. burgdorferi*, we defined two metrics, which we call the *cross-skew* and *dot-skew* (see Methods). The cross-skew metric reflects the orthogonality and magnitude of the vectors, and thus is expected to be particularly strong for *B. burgdorferi*. The dot-skew metric emphasizes the collinearity of the vectors. We computed the cross-skew and dot-skew for *B. burgdorferi*: cross-skew = 0.0541, dot-skew = 0.0083. For other species within the Borrelia and Borreliella genera, these respectively ranged from 0.0509 to 0.0727 and from 0.0044 to 0.0314. These cross-skew values are much larger than observed for other bacteria, as detailed below. In contrast, we computed much smaller cross-skew values for *S. thermophila* (0.0051), *M. tuberculosis* (7.2 10^− 5^), *F. periodonticum* (− 0.0028) and *A. phagocytophilum* (0.0089), reflecting their small magnitude skew vectors and/or their angles.

### Learning from thousands of genomes

**Fig. 3:**
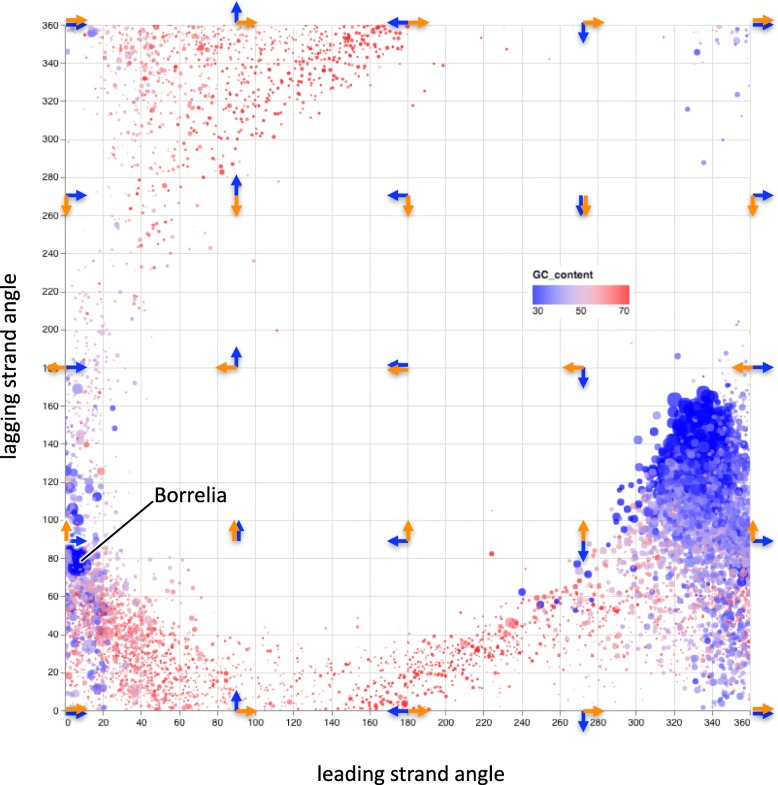
Relationship between the leading-strand angle and the lagging-strand angle for 7738 bacterial genomes. Colors denote GC content, ranging from low (blue) to high (red). Point sizes are proportional to the product of the magnitudes of the leading-strand and lagging-strand vectors. Overlaid arrows denote the interpretation of the angles as in Fig. 1 (blue = leading strand, orange = lagging strand)

We computed characteristic skews, angles and skew metrics for 7738 bacterial genome assemblies (see Methods, Additional file 2: Table S1). We observed genomes with strong skews and with negligible skews, at all possible angles between the characteristic skew vectors. We also created a web interface for generating species-specific skew plots and exploring their skew metrics, available at [28]. Visualization of these species-specific parameters demonstrates the wide diversity of bacterial genome composition. We compared the angles of the characteristic skew vectors (Fig. 3) and found that both tend to be constrained in low-GC bacteria (blue circles in Fig. 3, upper panels in Additional file 1: Figure S4), while they can present all possible values in high-GC bacteria (red circles in Fig. 3, lower panels in Additional file 1: Figure S4). Nevertheless, we found that the combination of these two angles is highly constrained: there is a clear avoidance of a large range of possible angular combinations in which the leading-strand and lagging-strand angles are both simultaneously in the range [90°..270°]. Of the few species that display these combinations, most have strong discrepancies between their annotated and computed origins of replication (Additional file 1: Figure S5).

**Fig. 4:**
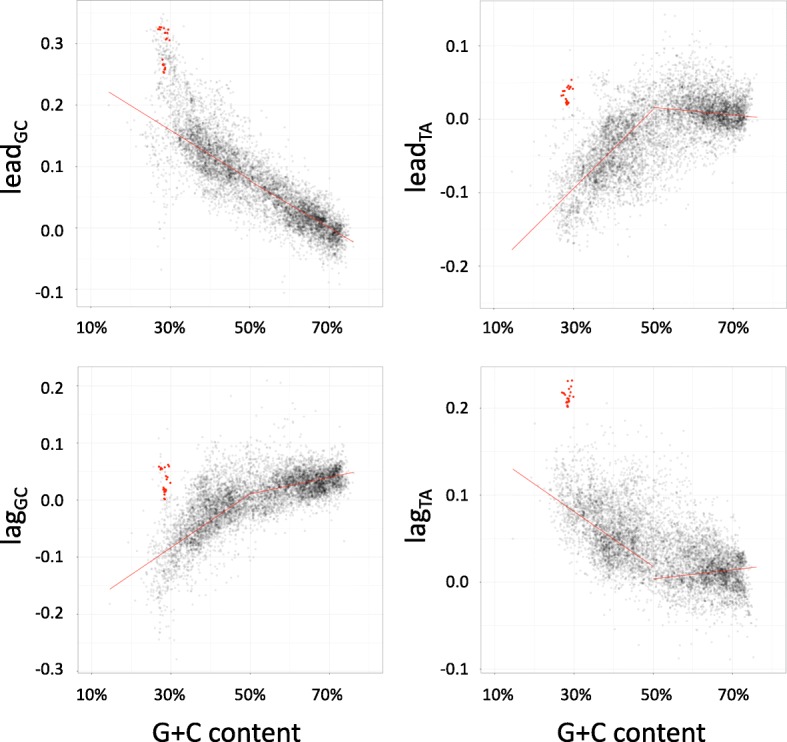
Relationship between the four characteristic skew values and G + C content, for 7738 bacterial genomes, highlighting Borreliaceae species (red points). Red lines represent robust regression lines computed by least quantile of squares method

**Fig. 5:**
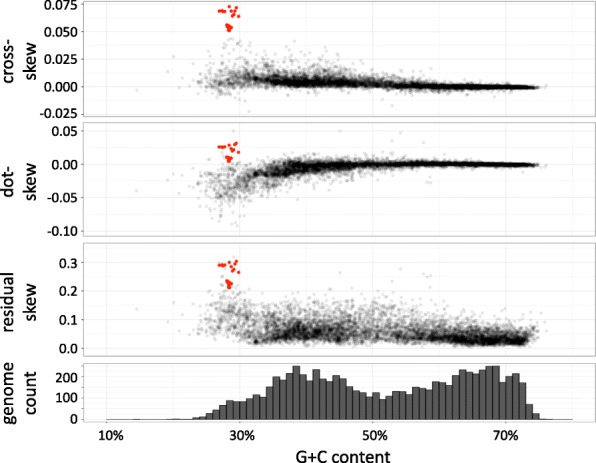
A: Skew metrics vs. G + C content for 7738 bacterial genomes, highlighting Borreliaceae species (red points). From top to bottom: cross-skew, dot-skew, residual skew, and histogram of number of species studied

We compared the four characteristic skews of 7738 bacterial genome assemblies with their corresponding G + C content (Fig. 4). We observed that all four skews are correlated with G + C content, and largely decrease in absolute value with increasing G + C content. While the mechanism behind this remains unclear, it could be largely explained by the high frequency of cytosine deamination experienced by many bacteria [20, 29]. Single-stranded DNA is susceptible to oxidative damage during both replication and transcription, leading to cytosine deamination and increased occurrence of C → T mutations. Replication-related mutations appear on the leading strand and transcription-related mutations occur on the coding strand, which is the leading strand for most bacterial genes [30]. This produces GC skew values that are higher (typically: larger positive values) on the leading strand and lower (typically: larger negative values) on the lagging strand, TA skew values that are lower (more negative) on the leading strand and higher (more positive) on the lagging strand, and lower overall G + C content. These trends are all observed here. However, if there are a significant number of genes oriented with the coding strand on the lagging strand, and if the level of transcription-related mutation is high, outliers may occur. This is also observed here for a small number of genomes at the low end of the G + C scale. As previously reported ([6, 22], Fig. 4), the relationships between skews and G + C content are different for bacterial genomes with low vs. high G + C content. We also observed a largely bimodal distribution of G + C content among sequenced bacterial genomes (Fig. 5, lower panel). We therefore fitted lines to the characteristic skews separately for bacterial genomes below and above 50% G + C content, and computed the deviations from the expected skews for each bacterial genome assembly. We used the R function MASS:lqs(), the leading robust linear regression method, to ensure that these lines accurately reflected the typical pattern, ignoring outliers.

The lead_GC_ and lag_TA_ values of Borreliaceae genomes are large and are clear outliers relative to the entire data set of 7738 genomes (Fig. 4). On the other hand, while the Borreliaceae lead_TA_ and lag_GC_ are close to zero and are not outliers relative to the entire data set, they are unusual for bacterial species with low G + C content, which tend to have negative values for these characteristic skews (Fig. 4). Thus, Borreliaceae genomes are unusual for all four characteristic skews. The deviations of characteristic skews for *B. burgdorferi* from the skews predicted by the fitted lines at the G + C content for *B. burgdorferi* are 0.091, 0.120, 0.106 and 0.124 for lead_GC_, lead_TA_, lag_GC_ and lag_TA_, respectively. Borrelia species that cause relapsing fever have even larger deviations from the expected values.

### Three novel metrics for analyzing genome skews

We described above several parameters for quantifying skews in individual bacterial genomes: the four characteristic skews and the magnitudes and angles of the vectors they define. Using these parameters, we defined two interrelated metrics for comparing and contrasting the skews of leading strand vs. lagging strand genes: the cross-skew and the dot-skew (see Methods). Furthermore, taking advantage of the availability of many thousand bacterial genome assemblies, we estimated expected values for each characteristic skew, as a function of the G + C content. We then used the observed deviations from these expected values to define a third metric: the *residual skew*.

We computed these three metrics for 7738 bacterial genome assemblies (available at [28]) and evaluated their relationship with G + C content (Fig. 5). All three are more independent of G + C content than the four characteristic skews; however, a large number of low G + C taxa have significantly negative dot-skew. Since G + C is not necessarily correlated with metabolism or lifestyle, these statistics may be more closely related to metabolism or lifestyle. For high G + C content bacteria, we observed that the cross-skew and the dot-skew are much more constrained than for lower G + C content species; these two metrics are most diverse for bacterial genomes under ~ 35% G + C. Compared to these two metrics, the residual skew is more diverse for all levels of G + C content. Borreliaceae genomes are clear outliers for all three metrics.

We compared dot-skew, cross-skew, and residual skew across 10 large clades on the same species discussed in [4] (Additional file 1: Figure S7). Dot-skew and cross-skew values were clustered at zero for the largest and most G + C diverse clades (e.g., Proteobacteria, Firmicutes); dot-skew clusters below zero only for certain very low G + C groups (Tenericutes, Thermotogae). Clade therefore provided little ability to explain dot- or cross-skew. In contrast, residual skew had a broader distribution within each clade, and clade-specific typical values. In contrast to results reported from the Z-curve correlation metric [22], which observed similar skews in Firmicutes, Tenericutes, and Thermotogae relative to other large clades, including Proteobacteria, dot-skew and cross-skew distributions for Proteobacteria are highly similar to their distributions in Firmicutes, and overlap significantly with Tenericutes and Thermotogae.

### The landscape of bacterial skews

**Fig. 6:**
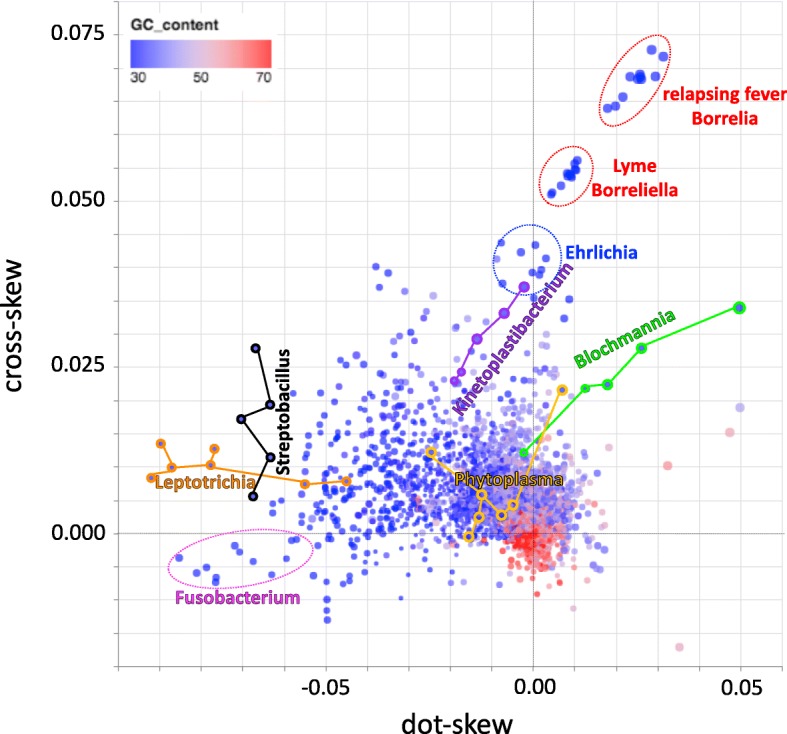
Integration of skew metrics (cross-skew vs. dot-skew, point size represents residual skew) for 7738 bacterial genomes, highlighting some genera of interest. All genomes colored by G + C content, ranging from low (blue) to high (red)

Finally, we combined all three metrics to generate a map of genome skews for all bacterial genomes (Fig. 6). In this map, most high G + C content bacteria are restricted to near the origin, while low G + C content bacteria show a more diverse spread.

We identified outlier taxa for each skew metric independently at a 1% significance level (see Methods, Additional file 1: Figures S8, S9; identified taxa are listed in Additional file 3: Table S2). Even at this stringent significance level, 1666/7738 (21.5%) of all taxa were considered outliers with negative dot-skew values, with a clear trend toward more negative dot-skew with decreasing G + C content for a subset of taxa. In summarizing the relationship between genus and skews (Additional file 3: Table S2a), we excluded outliers with negative dot-skew from consideration, and focused on association between genus and outliers of any other type.

The most extreme outliers observed are Borreliaceae, particularly the group of Borrelia genomes that cause relapsing fever. Borreliaceae species have a low G + C content genome suggesting an increased indifference towards GC rich codons and thus energetically cheaper amino acids. This is further supported by the fact that they lack amino acid and nucleotide synthesis pathways. The observed strand specific nucleotide usage and skew patterns in these bacteria may thus be driven by a relaxation of the selection for energy efficiency [4]. We computed selection coefficients for the genomes discussed in [4] and provide plots of these values against dot- and cross-skews (Additional file 1: Figures S10, S11). For Borreliaceae, we observe relaxation of selection on TA biases on the lagging strand.

Our finding on the difference in genome skew metrics among the relapsing fever and the Lyme causing species could be associated with their unique ecological and vector-related traits such as the variety of vectors, speed of transmission to a new host and maintenance in nature by transovarial transmission. For example, the vectors for the relapsing fever Borrelia species represent a variety of arthropods while for the Lyme disease causing group, the widely known competent vectors are certain species of the prostriate genus Ixodes [31]. In comparison to the Lyme disease spirochetes, most relapsing fever Borrelia are efficiently transmitted to the host soon after feeding by the tick begins [31, 32]. In addition, most species in the relapsing fever Borrelia group exhibit transovarial transmission in their tick vectors [31]. Genomes in the genus Ehrlichia (see example in Fig. 4) are also outliers in all three metrics and show similar skew values as Borreliella genomes. Ehrlichia are intracellular vector-borne pathogens of vertebrates; like Borrelia, they have diminished biosynthetic abilities [33]. Ehrlichia are in the Rickettsiales order and are phylogenetically unrelated to Borreliaceae; the genome of *Ehrlichia canis* has a single circular chromosome and no plasmids [34]. Multiple other genera became evident as outliers of interest, discussed below.

## Discussion

We have devised three novel metrics to study bacterial genome composition biases, integrating knowledge of the nucleotide skews in annotated genes, the direction of transcription relative to replication, and the G + C content of the genome.

The first two metrics (cross-skew and dot-skew) are computed based on an individual genome’s characteristic skew vectors, and they quantify the strength and relationship between the mutation and selection pressures on genes on the leading vs. lagging strands. Since the two metrics depend on the magnitudes of these vectors and *the angle between them*, identical skew metric values might be computed for bacterial species that differ, for example, in leading strand angle while maintaining a constant angle between the two vectors. We found this to be the case only for bacteria with very small skews, leading to cross-skew and dot-skew values near zero for all possible leading strand angles (Additional file 1: Figure S6).

Strong and positive dot-skew values (Fig. 6, right) indicate similar compositional constraints on all genes, relative to the direction of replication; an example of this pattern is observed in the obligate intracellular parasite *Chlamydia pneumoniae* [35] (Fig. 2). Conversely, strong and negative dot-skew values (Fig. 6, left) reflect opposite compositional constraints on leading and lagging strand genes (i.e., transcribed in the same or opposite direction as they are replicated); extreme examples of this pattern are observed in fusobacteria including *Fusobacterium periodonticum* [36], *Leptotrichia buccalis* [37], and *Streptobacillus moniliformis* [38], the causal agent of rat bite fever. Positive dot-skew values can thus be interpreted as reflecting constraints driven mostly by the replication process, while negative dot-skew values largely reflect transcriptional and translational constraints that arise from preference for nucleotides and amino acids that are energetically cheaper to synthesize [4].

The cross-skew quantifies the strength and orthogonality of the compositional skew vectors for leading and lagging strand genes. Genomes with high cross-skew values (Fig. 6, top) demonstrate skew patterns inconsistent with purely replicational or transcriptional constraints; Borreliaceae and Ehrlichia species are prime examples of this pattern. Borreliaceae and Ehrlichia species lack amino acid and nucleotide synthesis pathways; the observed elevated cross-skew values in these pathogens may thus reflect a relaxation of the selection for energy efficiency that drives nucleotide usage and thus skews [4], possibly combined with more complex constraints imposed by a life cycle that involves recurring transitions between mammalian and invertebrate (tick) hosts. We observed similar skew patterns in Kinetoplastibacteria (Fig. 6), which are endosymbionts of insect-infecting trypanosomatid flagellates [39] with multiple biosynthetic adaptations to life in the intracellular environment. Likewise, we observed distinct skew patterns among Blochmannia species (Fig. 6); these are also intracellular endosymbionts that lost multiple biosynthetic pathways and rely on the metabolic machinery of their carpenter ant hosts [40]. Other groups with a more modest, but significant enrichment for high cross-skew include other host-associated anaerobes such as Bacteroides, Lachnospiraceae, Eubacterium spp., and other Clostridiales, and extremophiles such as Thermoanaerobacter, Thermosipho, and Thiomicrospira. In contrast, heavily sampled clades that appear to have fewer than expected cross-skew, residual skew, or positive dot-skew outliers include Actinobacteria (Streptomyces, Mycobacterium, Corynebacterium, Nocardia), γ-Proteobacteria (Pseudomonas, Vibrio), and Bacilli (Bacillus, Lactobacillus, Streptococcus, Paenibacillus).

The third metric (residual skew) capitalizes on the current availability of thousands of complete or draft bacterial genomes to empirically assess how unusual a genome’s skews are relative to the expected values as learned from other genomes. This analysis, which has not been possible until recent times, revealed that bacterial genomes with low G + C content typically have negative TA skews in leading strand genes and negative GC skews in lagging strand genes, and that these negative skews increase in magnitude as G + C content decreases (Fig. 4). On the background of these trends, the weakly positive skews observed in Borreliaceae species are highly unusual. This pattern is not evident relative to the global collection of genomes since the weakly positive Borreliaceae skews are comparable to those observed in high G + C content bacteria.

The presence or absence of chromosomal maintenance pathways can shape genome composition skews. Outlier genomes such as *B. burgdorferi, Candidatus Kinetoplastibacterium crithidii, Ehrlichia chaffeensis, Buchnera aphidicola,* and *Blochmannia floridanus*, have reduced genomes with more limited repair mechanisms. Yet the major pathways are all present, even if they tend to contain fewer genes than bacteria such as *Escherichia coli* and *Yersinia pestis* (see Additional file 4: Table S3). No single gene or simple combination of genes defines the outliers. The outliers do lack mismatch-repair *mutH*, recombination *lexA*, and base excision repair *mug*, but so do non-outlying genomes such as *Francisella tularensis*. Our regression analysis quantifies these deviations from expectation and integrates them into a unified metric that highlights the unusual skews in Borreliaceae species (Fig. 5) and also identifies other species as having skew patterns that are significantly unusual relative to the bulk of bacterial species. Of particular note are Phytoplasma species (Fig. 6); these are intracellular pathogens of multiple plant species that use insects as transmission vectors [41, 42], in similarity to Borreliaceae and Ehrlichia for mammals.

Through analysis of all genic regions of any conservation level, our metric measurements could accurately predict and/or support taxonomical distinctions among closely related genomes with shared biological and genetic features. An example is the Lyme-causing and relapsing fever groups of spirochaetes that have long belonged to the same genus Borrelia. The two groups have just recently been split into two distinct genera [31].

## Conclusions

We described here three novel metrics for quantifying bacterial genome composition skews and presented examples of their application to identify bacterial species with unusual skew patterns. Our metrics take advantage both of information about the genome of a single species and of patterns discernable from studying genomes of thousands of species - even those not yet finished and fully annotated. While some of the genera identified as skew outliers are phylogenetically close (e.g., Fusobacterium, Streptobacillus and Leptotrichia), our metrics identified similar skew patterns in genera of bacteria that are phylogenetically unrelated, like Borrelia, Ehrlichia and Kinetoplastibacterium, and (when considering the residual skew) Phytoplasma. These very disparate bacterial species share lifestyle characteristics (intracellularity and transmission via insect vectors), suggesting that our novel metrics successfully capture effects on genome composition of biosynthetic constraints and of interaction with the hosts.

## Additional files

Additional file 1: Supplementary figures. (DOCX 1778 kb)

Additional file 2: **Table S1.** Accession, name, GC content, skew parameters and skew metrics for 7738 bacterial genomes studied. (XLSX 1838 kb)

Additional file 3: **Table S2.** (a) Summary of outliers counted by genus. (b-d) Lists of 7738 bacterial genomes sorted by dot-skew, cross-skew, and residual skew, respectively, with outliers indicated in red (positive outlier) or blue (negative outlier). (e) Summary of the derivation of the statistical thresholds. (XLSX 1059 kb)

Additional file 4: **Table S3.** Presence and absence of genes associated with replication, recombination, and repair. Data were retrieved from the KEGG Orthology database [45] for homologous recombination (ko03440), mismatch repair (ko03430), DNA repair and recombination (ko3400), nucleotide excision repair (ko03420), and base excision repair pathways (ko03410). Asterisks denote skew outliers. (XLSX 9 kb)

## Abbreviations

GFF: General Feature Format
NCBI: National Center for Biotechnology Information
ori: Origin of replication
RMSD: Root mean square deviation
ter: Replication terminator site
