# Supplementary material for: Novel metrics for quantifying bacterial genome composition skews
Source: BMC Genomics. 2018 Jul 11;19:528. doi: 10.1186/s12864-018-4913-5 (PMC6042203; doi:10.1186/s12864-018-4913-5)

**Supplementary Figures**

**Novel metrics for quantifying bacterial genome composition skews**

Lena M. Joesch-Cohen, Max Robinson, Neda Jabbari, Christopher Lausted, Gustavo Glusman*

*** Correspondence:** Gustavo Glusman: Gustavo@SystemsBiology.org

**Figure S1.** Concordance between the location of origins of replication as annotated in the DoriC database, with the locations computed using the GC disparity rule. Most species have discrepancy <0.1 (i.e., <10% of chromosome length). Green points: species with discrepancy between 0.1 and 0.25. Red points: species with discrepancy >0.25.


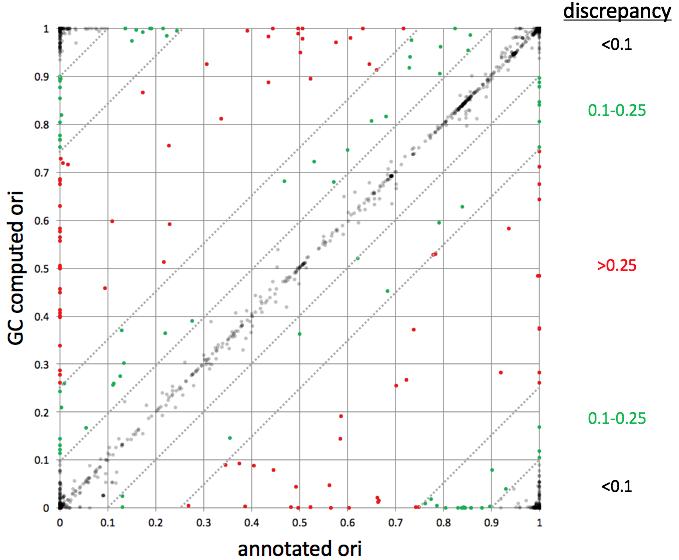


**Figure S2.** Robustness of skew metrics to incompleteness of genome assembly. For each of *B. burgdorferi* (left) and *E. coli* (right), we simulated up to 100 genome cuts leading to progressive loss of sequence. Top graphs: the amount of genomic sequence in the surviving contigs for each simulation. Bottom graphs: the resulting cross-skew and dot-skew estimates.


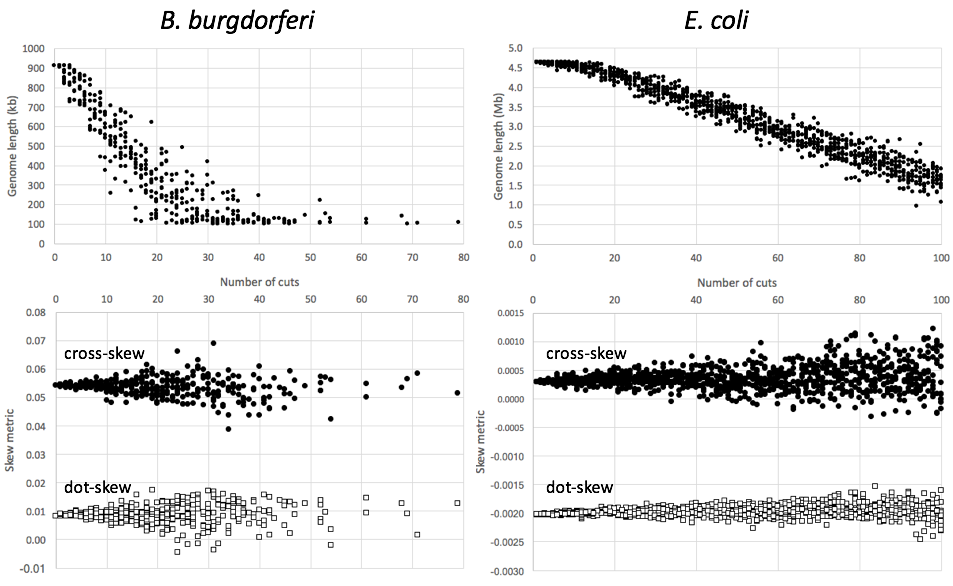


**Figure S3.** Estimates of skew parameters and metrics vs. amount of sequence included simulations of genome drafts for *B. burgdorferi* and *E. coli*. Whisker plots represent median values, quartiles, minima, maxima and outlier values.


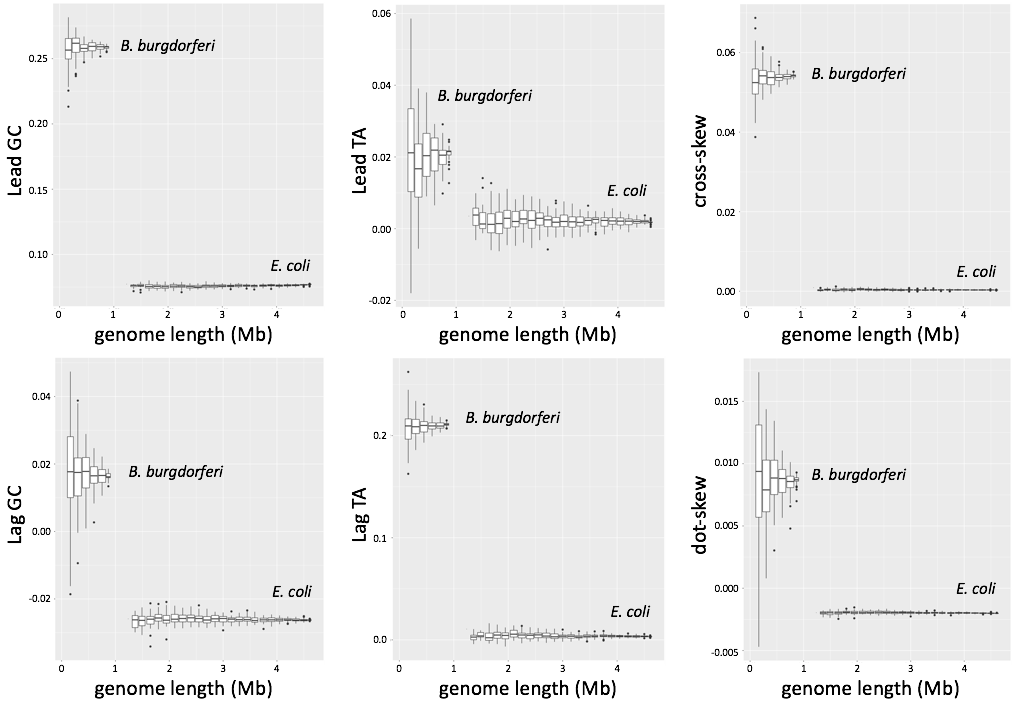


**Figure S4.** Comparison of the leading-strand angle and the lagging-strand angle for 7738 bacterial genomes, highlighting GC content ranges from low (blue) to high (red).


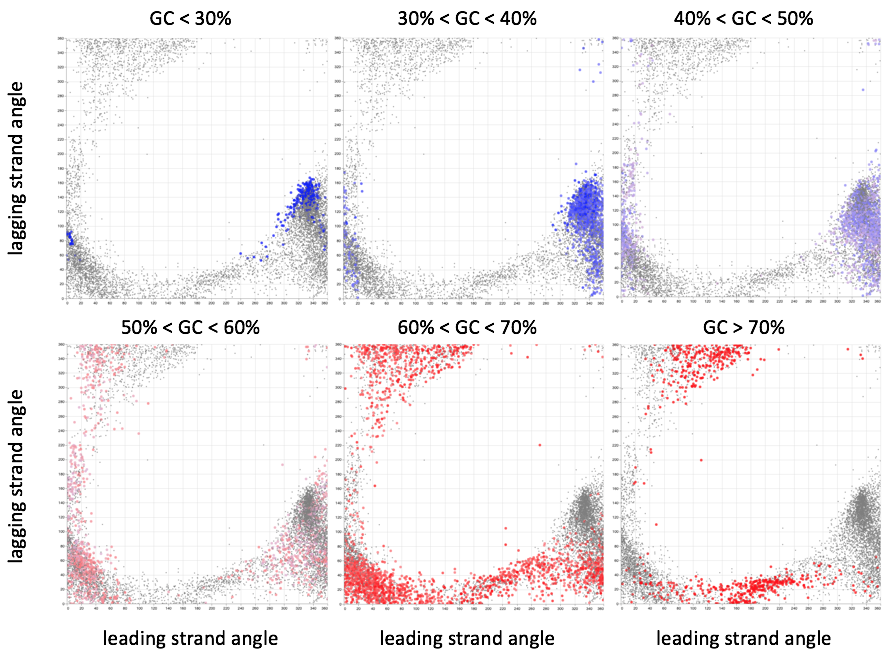


**Figure S5.** Comparison of the leading-strand angle and the lagging-strand angle for 7738 bacterial genomes, highlighting species with moderate (green) or high (red) discrepancy between the annotated locations of origins of replication and the locations of the same based on the GC disparity rule.


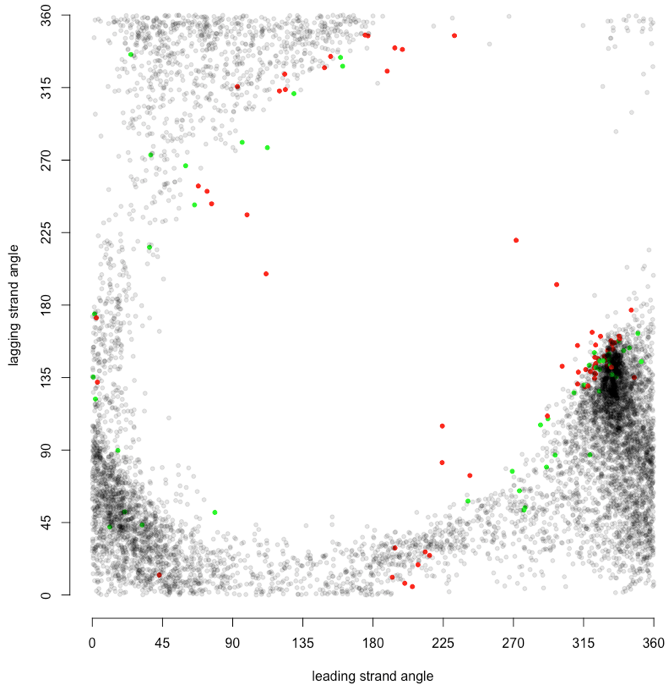


**Figure S6.** Left-side plots: cross-skew, dot-skew and residual skew vs. the leading-strand angle for 7738 bacterial genomes. Right-side plot: Comparison of the angle *θ* between the characteristic vectors and the leading-strand angle for 7738 bacterial genomes. Color coded by GC content from low (blue) to high (red). Circle area represents the product of the magnitudes of the characteristic skew vectors.


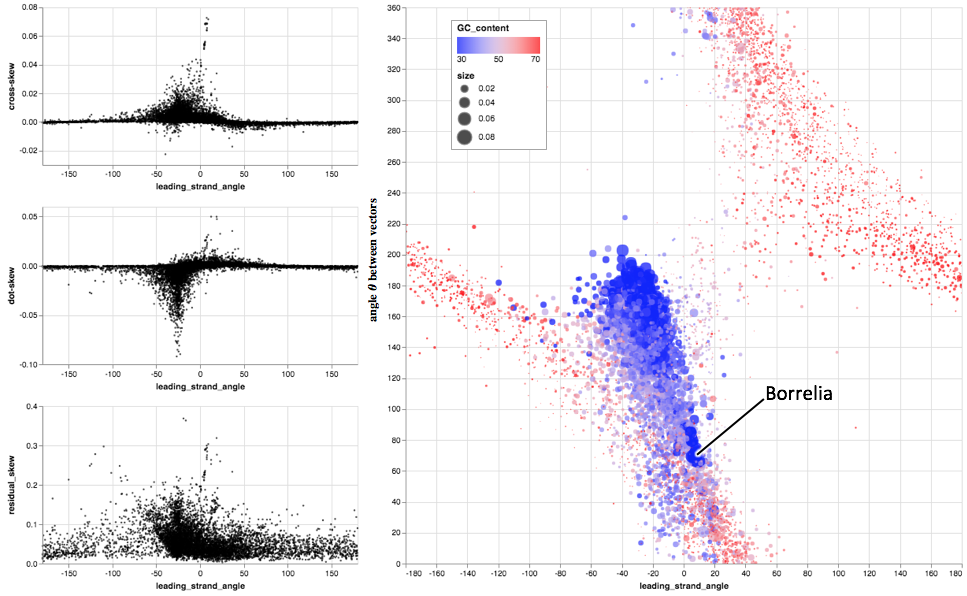


**Figure S7.** Distribution of skew metrics for ten large clades of bacteria: Actinobacteria, Bacteroidetes/Chlorobi, Chlamydiae/Verrucomicrobia, Cyanobacteria, Deinococcus/Thermus, Firmicutes, Proteobacteria, Spirochaetes, Tenericutes and Thermotogae.


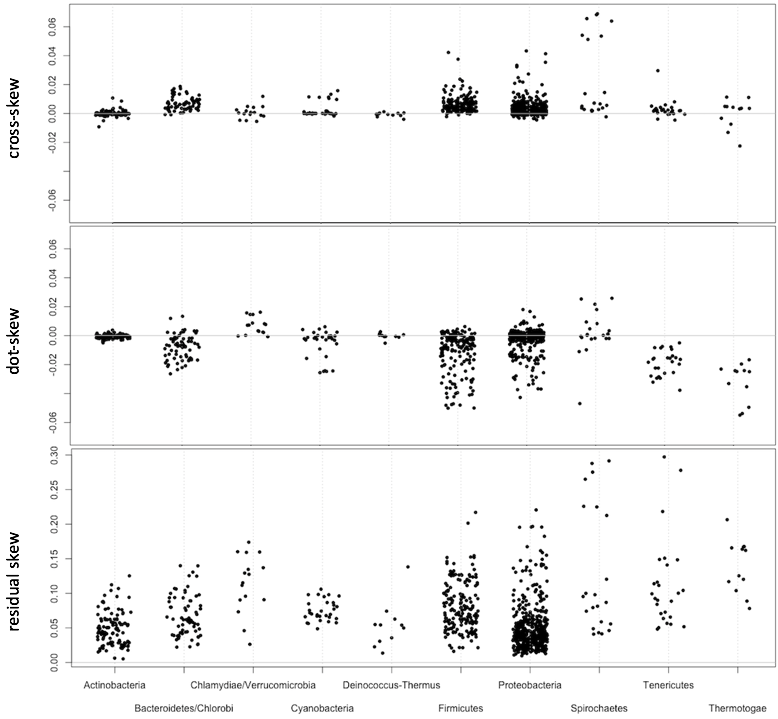


**Figure S8.** Determination of outliers for the cross-skew and dot-skew metrics by MAD-Median rule.


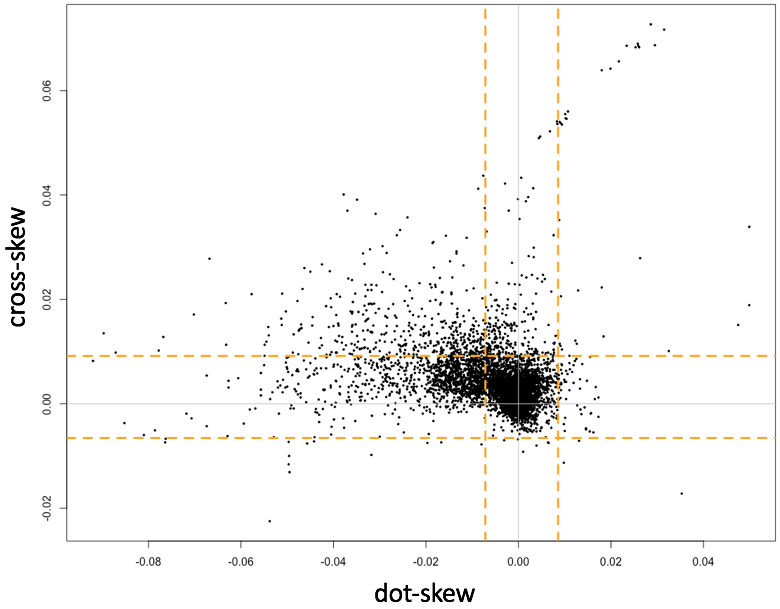


**Figure S9.** Determination of the outlier threshold for the residual skew metric by quantile-quantile plot with a simulated model, (100x sample to reduce threshold variance).


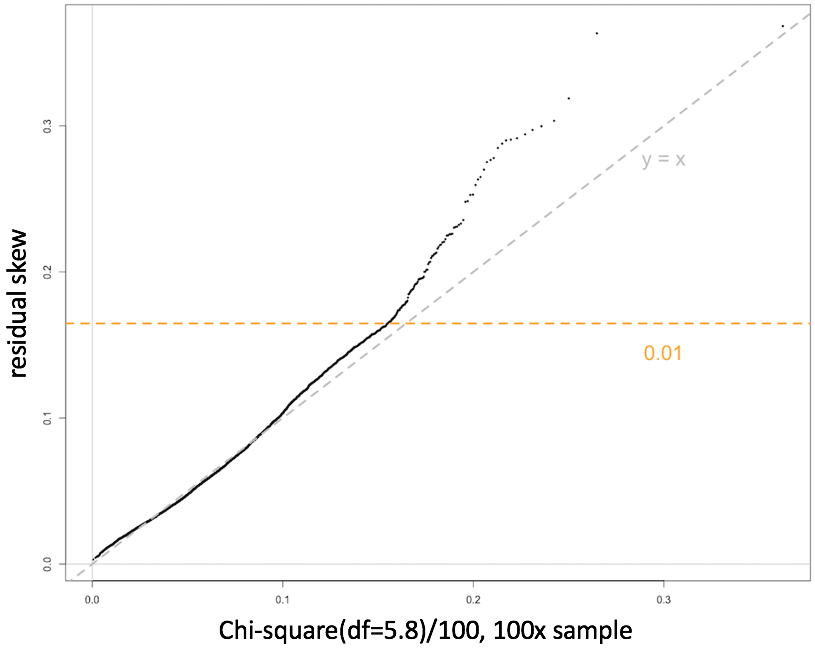


**Figure S10.** Selection coefficients vs. dot-skew values for GC and AT on the leading and lagging strands for 875 genomes, highlighting Borreliaceae species (red points).


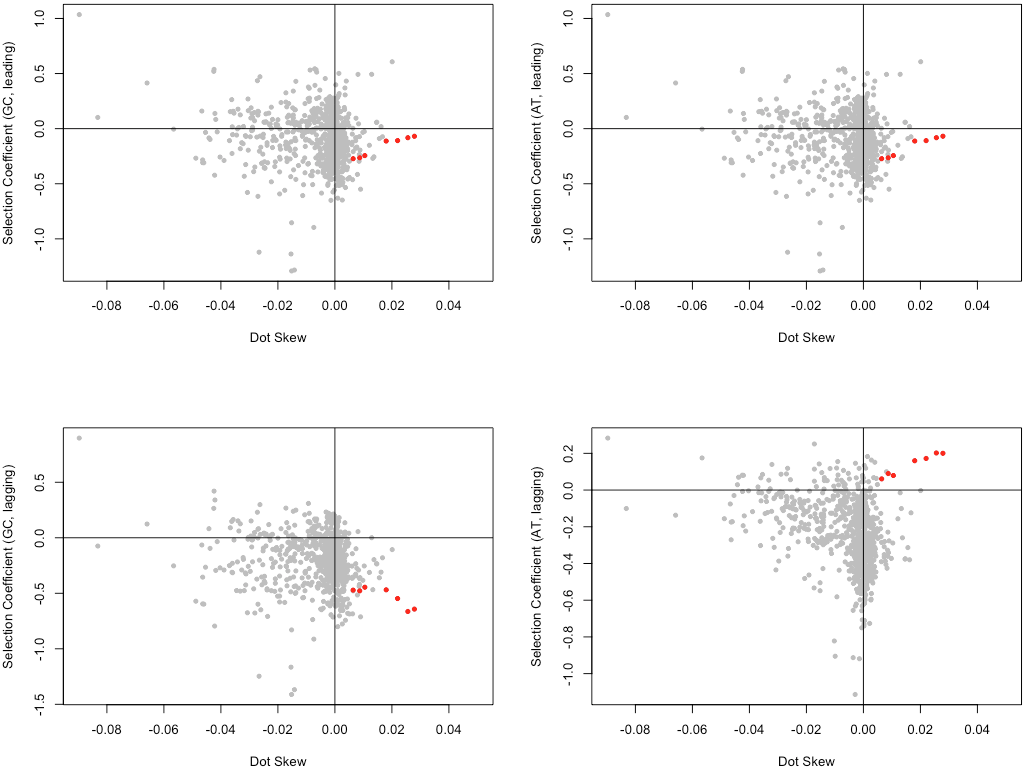


**Figure S11.** Selection coefficients vs. cross-skew values for GC and AT on the leading and lagging strands for 875 genomes, highlighting Borreliaceae species (red points).


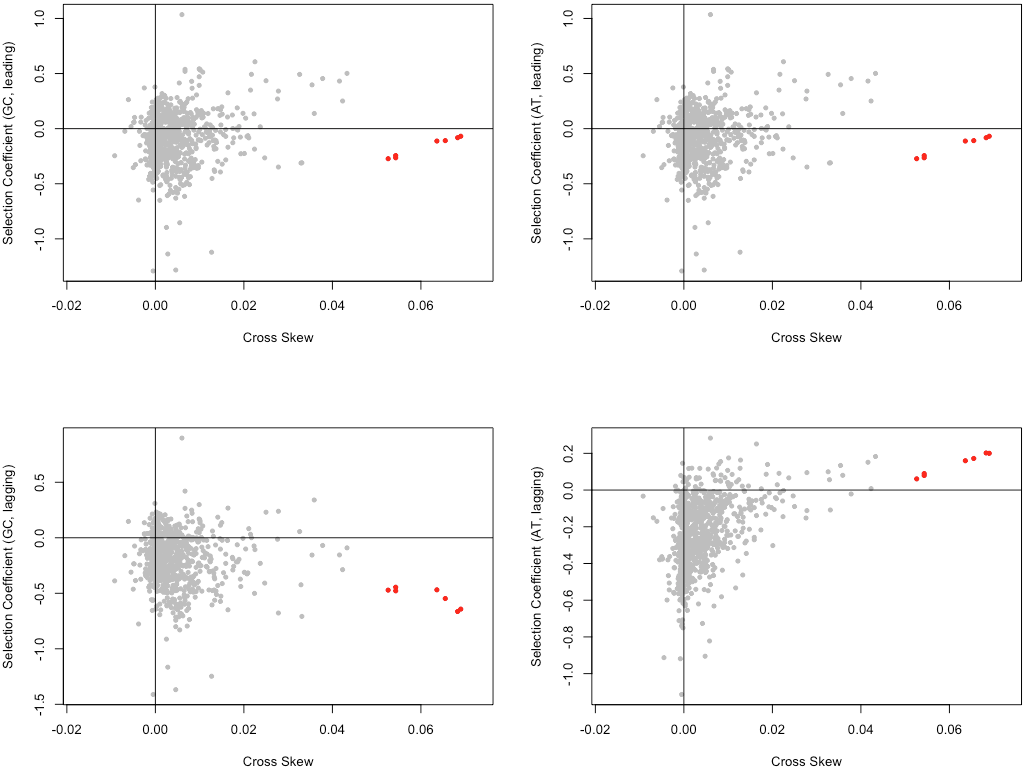

Supplement: Supplementary file 1 — Supplementary figures. (DOCX 1778 kb) [file 12864_2018_4913_MOESM1_ESM.docx]
